# Harnessing Particle Size Segregation To Tune Molecular Additive Distribution in Coatings

**DOI:** 10.1021/acs.iecr.5c04051

**Published:** 2026-01-06

**Authors:** Huyen Le, Timothy J. Murdoch, Aitor Barquero, Radmila Tomovska, Ignacio Martin-Fabiani

**Affiliations:** † Department of Materials, 5156 Loughborough University, Leicestershire LE11 3TU, U.K.; ‡ POLYMAT and Departamento de Química Aplicada, Facultad de Químicas, UPV/EHU, Joxe Mari Korta Zentroa, University of the Basque Country, Tolosa Hiribidea 72, Donostia-San Sebastian 20018, Spain; § IKERBASQUE, Basque Foundation for Science, Maria Diaz de Haro 3, Bilbao 48013, Spain

## Abstract

The spatial distribution of small-molecule additives within polymer coatings plays a critical role in determining their performance, from antimicrobial activity to corrosion resistance. While size segregation during film formation has been harnessed to control the distribution of nanoparticles or polymers, its potential for controlling the molecular additive distribution remains largely unexplored. Here, we investigate how bimodal colloidal blends can direct the positioning of a model additive, nickel­(II) phthalocyanine (NiPc), during drying. Using complementary microscopy and spectroscopy techniques, we show that NiPc predominantly associates with the smaller particles in the blend due to their larger total surface area. At low and medium relative humidities, this leads to an enrichment of NiPc at the film’s surface via small-on-top stratification. Slow drying at high humidity results in additive accumulation near the substrate due to aggregation and sedimentation of small particle clusters. Release studies reveal that bimodal films generally slow the initial burst release of NiPc compared to monomodal controls, enabling more sustained delivery over time. Fitting release profiles with the Korsmeyer–Peppas model confirmed Fickian diffusion as the dominant mechanism, with differences in pore structure potentially influencing diffusion rates. Overall, our findings demonstrate that particle size distribution and evaporation rate can be tuned to modulate the location and release behavior of molecular additives in coatings. This approach provides a versatile route for designing functional films with tailored performance with potential applications in medical, marine, and protective technologies.

## Graphical abstract

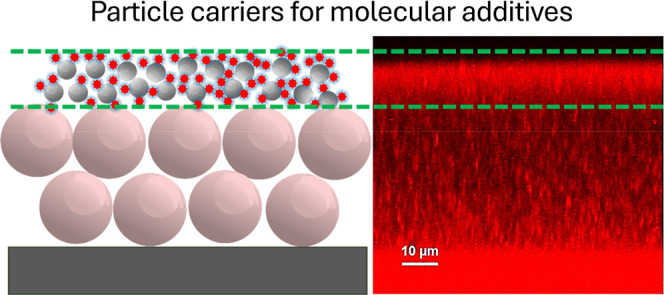

## 1 Introduction

The way an additive is distributed within a coating strongly influences its performance and properties. Some additives are most effective when concentrated at the top surface of the coating, such as biocides^1^ or abrasion-resistant agents.^2^ Others function best when they are evenly dispersed throughout the bulk of the coating, for example, plasticizers^3^ or opacifiers.^4^ In contrast, certain additives deliver optimal performance at the coating–substrate interface, such as adhesion promoters.^5^ For these reasons, controlling the distribution of additives in coatings is an ongoing quest for academics and industrialists alike.

In recent years, the phenomenon of size segregation in drying coating formulations has been proposed as an approach to control ingredient distribution. Studies have demonstrated the self-stratification of bimodal blends of large and small polymer particles, where small-on-top stratification can occur when evaporation is dominant over diffusion for both particle populations.^6–9^ The same method has been used in blends of polymer particles with functional nanoparticles, enriching the top layer content with the latter to enhance the antibacterial^10,11^ or abrasion resistance^2^ performance of the resulting coatings. Recent works have investigated the influence of rheology modifiers in the self-stratification process, including both associative^12^ and nonassociative^13^ thickeners. Notably, the size segregation phenomenon also occurs when polymer particles and chains are blended, with the effective size ratio being the diameter of the particles over that of the radius of gyration of the polymer.^14^ Surprisingly, very few studies have harnessed this size segregation process to control the distribution of molecular additives in coatings. In the only relevant study we are aware of,^15^ Römermann and Johannsmann tuned the horizontal and vertical distribution of cross-linkers in drying latex dispersions via changes in the polymer glass transition temperature and the concentration of an associative thickener. This led to a situation of arrest of the polymer particles, and therefore, the differences in distribution observed were mostly related to the diffusion and convection of the cross-linkers in the weak gel matrix.

In the present study, we chose nickel­(II) phthalocyanine (NiPc) as a model for a small-molecule additive with limited solubility in water. Additives of this nature are likely to adsorb strongly at surfaces and interfaces and, therefore, be influenced by particle size segregation processes taking place in the formulation. Moreover, NiPc is a fluorophore and, therefore, enables the imaging of its distribution within the film. From an application point of view, although we did not test that in this study, NiPc is a photosensitizer which can convert oxygen into reactive oxygen species (ROS) known to damage bacterial membranes.^16^

Herein, we investigate how particle size segregation in drying binary colloidal blends can be harnessed to control the spatial distribution of molecular additives in polymer coatings. Using NiPc as a model additive, we combine advanced imaging and analytical techniques to track its localization under different drying conditions. Although NiPc shows no intrinsic preference for either particle type, the significantly larger specific surface area of the small particles makes them the dominant carriers of the additive. Our results demonstrate that the interplay between particle size ratio, evaporation rate, and additive–particle interactions governs both the microstructure of the coating and the final positioning of NiPc across the film thickness. We show that fast and medium drying conditions promote small-on-top stratification, leading to NiPc enrichment at the air–film interface, whereas slow drying facilitates aggregation and sedimentation of small particle clusters that transport NiPc toward the substrate. These distinct microstructures have measurable consequences for additive release in the presence of water, with bimodal films generally suppressing the initial burst release and enabling more sustained delivery compared to monomodal control films. These insights provide a pathway to tailor the performance and release behavior of functional small molecules in coatings with potential applications in antimicrobial, medical, and protective technologies.

## 2 Materials and Methods

### 2.1 Materials

#### 2.1.1 Latex Synthesis

Emulsion polymerization was used to synthesize two latex dispersions with two different particle sizes, which we will refer to as large and small from now on. Both latexes were composed of poly­(butyl acrylate-*co*-methyl methacrylate) (P­(BA-*co*-MMA)). The large latex particles obtained through surfactant-free seeded emulsion polymerization, a BA/MMA mass ratio of 50:50, and 1 wbm % sodium styrenesulfonate (NaSS) as a stabilizer, as described by Bilgin et al.^17^ The initiator used in the synthesis of the large particles was a redox pair consisting of *tert*butyl hydroperoxide as an oxidant and Bruggolite FF7 as a reductant, in a 1:1 weight ratio and a total of 0.54 wbm %. The seed composition was MMA/BA 50/50. The small particles were synthesized using a BA/MMA mass ratio of 15:85, ammonium persulfate as an initiator, and sodium dodecyl sulfate (SDS, 10 wbm %) as a stabilizer in a microemulsion polymerization process.

Dimethyl sulfoxide (DMSO, purity ≥99.9%, Sigma-Aldrich), acetone (purity ≥99.5%, Sigma-Aldrich), and nickel­(II) phthalocyanine-tetrasulfonic acid tetrasodium salt (NiPc, Sigma-Aldrich) were used as received. All aqueous solutions were made with ultrapure water (18.2 MΩ cm^–1^, Suez Select Fusion).

#### 2.1.2 Coating Preparation

Colloidal blends were prepared to ensure that the particle volume fractions in the dry films for large and small particles were ϕ_large_ = 0.7 and ϕ_small_ = 0.3, respectively. The sample preparation procedure began by diluting the stock dispersions of large and small particles to a 10 wt % solids content and blending them in appropriate amounts. The resultant large-small latex dispersions were subjected to vortexing for 30 s at a speed of 2400 rpm. Then, a small amount of NiPc stock solution in DMSO (10 wt %) was added to the latex dispersions to reach NiPc concentrations of 0.1, 0.5, and 1 wt % in the dry film. Prior to casting, square glass coverslips (18 mm × 18 mm) were washed with acetone, dried with compressed air, and treated in an ultraviolet ozone cleaner (Ossila) for 10 min to enhance their hydrophilicity. Then, 200 μL of the colloidal blends were cast on the coverslips using a micropipette and left to film form at 25 °C and three different relative humidities (10%, 50%, and 90%) inside an environmental chamber (HCP105, Memmert). The corresponding evaporation rates were measured as described in the Supporting Information (see Figure S1 and associated text), yielding 2.12 × 10^–7^ m/s (fast), 8.47 × 10^–8^ m/s (medium), and 1.04 × 10^–8^ m/s (slow).

### 2.2 Atomic Force Microscopy

Topography maps of the surface of the dried films were acquired using a Bruker BioScope Resolve instrument (Bruker, Santa Barbara, CA) in ScanAsyst mode. All samples were imaged using silicon probes (Bruker, RTESPA-150) with a nominal spring constant of 5 N/m and a tip radius of 8 nm. For each sample, at least three different 3 μm × 3 μm regions were imaged to ensure that a truly representative image of the film surface was obtained. Images were analyzed using the NanoScope Analysis 2.0 software.

### 2.3 Confocal Fluorescence Microscopy

Cross-sectional NiPc distribution maps were obtained using an inverted time-resolved confocal microscope installed on an Olympus IX73 (PicoQuant MicroTime 200). The NiPc fluorophore was excited by using a 637 nm diode laser. A UPLSAPO60XW Olympus objective lens mounted on a piezo and a 50 μm pinhole were used to acquire 80 μm × 80 μm images with a 256 × 256 pixel resolution. Light emissions from the samples were detected by using a hybrid photomultiplier detector assembly (PMA). Images were processed using the SymPhoTime software (PicoQuant), and ImageJ analysis^18^ software was employed to obtain one-dimensional profiles of fluorescence intensity versus film height. A second-order polynomial correction was made to account for the depth dependence of the detected fluorescence intensity.

### 2.4 Differential Scanning Calorimetry

Glass transition temperatures, *T*_g_, of dried latex samples were measured using a DSC-Q2000 from TA Instruments. 3–5 mg of samples were placed in aluminum hermetic pans under nitrogen flow, cooled to −80 °C, and then heated to 150 °C, with a heat ramp rate of 20 °C/min. The glass transition temperature was determined by using the inflection point method and verified with the maximum of the first derivative.

### 2.5 Dynamic and Electrophoretic Light Scattering (DLS/ELS)

A Malvern Zetasizer Ultra (Malvern Panalytical, Malvern, UK) was used to carry out both DLS and zeta potential measurements. The 10 wt % latexes were diluted to 0.1 wt % with deionized water. Samples were illuminated by a 120 mW He–Ne laser at 633 and kept at 25 °C within a DTS070 folded capillary cell, using the backscattering angle for detection. All measurements were performed in triplicate. The Malvern ZS XPLORER software was used to analyze the data.

### 2.6 Scanning Electron Microscopy

Cross-sectional images of the coatings were acquired using a JEOL JSM-7100F field emission scanning electron microscope. Samples were prepared via freeze-fracture using liquid nitrogen. Samples were placed on vertical SEM sample holders and coated with a gold/palladium alloy to enhance the conductivity. Images were obtained using the secondary electron detector at a constant accelerating voltage of 5.0 kV and a probe current of 273.50 pA. The working distance was between 10 and 15 mm, depending on the size of the sample under study.

### 2.7 Ultraviolet–Visible (UV–Vis) Spectroscopy

Absorption spectra were determined using a UV–vis–NIR spectrophotometer (Cary 5000, Agilent) with tungsten halogen visible and deuterium arc UV light sources. Absorption values were measured across the wavelength range between 200 and 800 nm, using a 1 nm wavelength step size. The spectral bandwidth was 2 nm. Quartz cuvettes of a 10 mm optical path length and a 3500 μL total volume (100 macrocells, Hellma Analytics) were used. For the preferential absorption experiments, samples were centrifuged using a Himac FNX series micro ultracentrifuge at 120,000*g* for 60 min at 25 °C to sediment the latex particles together with any bound NiPc.

### 2.8 Release Testing

An investigation into the release behavior of NiPc from films formed under different environmental conditions was conducted through a series of soak test experiments using deionized water. 1 mL of deionized water was applied onto a sample, followed by liquid recovery and replenishment at 2 min intervals from *t* = 0 to *t* = 10 min and then at 10 min intervals up to *t* = 50 min. UV–vis measurements of the recovered liquid samples enabled the monitoring of NiPc release from the coatings using the Beer–Lambert law

A=ϵbc

where ϵ is the molar extinction coefficient, *b* is the optical path length, and *c* is the concentration. A calibration curve for NiPc dispersions in water yielded ϵ = 0.449 × 10^5^ M^–1^ cm^1^ at λ = 623 nm. See Figures S2 and S3 for more details. The measured Q-band (550–750 nm) presents two different peaks, indicating the coexistence of both NiPc aggregates and monomers in the water phase.^19^ The formation of these aggregates is driven by π–π interactions and is enhanced by the limited solubility of NiPc in water.

## 3 Results and Discussion

Table 1 contains the main physical characteristics of the two types of polymer particles that were blended in this study for forming films. It is important to note that the large particles have a glass transition temperature, which is below our film formation temperature (25 °C) and therefore are expected to coalesce and undergo interdiffusion. Small particles have a glass transition temperature above our film-forming temperature and thus are expected to retain their shape, facilitating their identification via the various microscopy techniques we used in this study. The DSC traces of the dried copolymers can be found in Figure S4.

**Table 1:** Characteristics of the Two Latexes Used in the Bimodal Lends: Hydrodynamic Diameter (*D*_H_), Zeta Potential (ζ), Polydispersity Index (PDI), and Glass Transition Temperature (*T*_g_)

latex	*D*_H_ (nm)^a^	ζ (mV)^b^	PDI^a^	*T*_g_ (°C)^c^
large	**267** **±** **2**	**–46.2** **±** **0.1**	**0.02** **±** **0.01**	**16.5**
small	54.1 ± 0.2	–35 ± 1	0.06 ± 0.02	41.9

a As measured by DLS.

b As measured by ELS.

c As measured by DSC.

### 3.1 Model Predictions

To make a prediction as to whether stratification will occur during the drying of our colloidal blends, we employ both the ZJD^9^ and Sear^13^ models. Péclet numbers of the small particles, *Pe*_S_, were calculated (see Supporting Information for details) for the three evaporation rates studied: 20 (10% RH), 8 (50% RH), and 1 (90% RH). According to the ZJD model, all our systems fulfill the condition α^2^(1 + *Pe*_S_)­ϕ_S_ > 1, and therefore, we should expect small-on-top microstructures in all our films (see Figure 1a). However, the prediction from the Sear model considers only the faster evaporation rate (lower RH) scenario as a candidate for small-on-top stratification. It is important to note that the ZJD model has been reported to overpredict stratification on multiple occasions,^20^ with actual stratification taking place at α, *Pe*_S_, and/or ϕ_S_ larger than those predicted. As we will show in the next section, the predictions from the Sear model are closer to what we observe experimentally, but there are caveats about the model, which will be discussed later.

**Figure 1:**
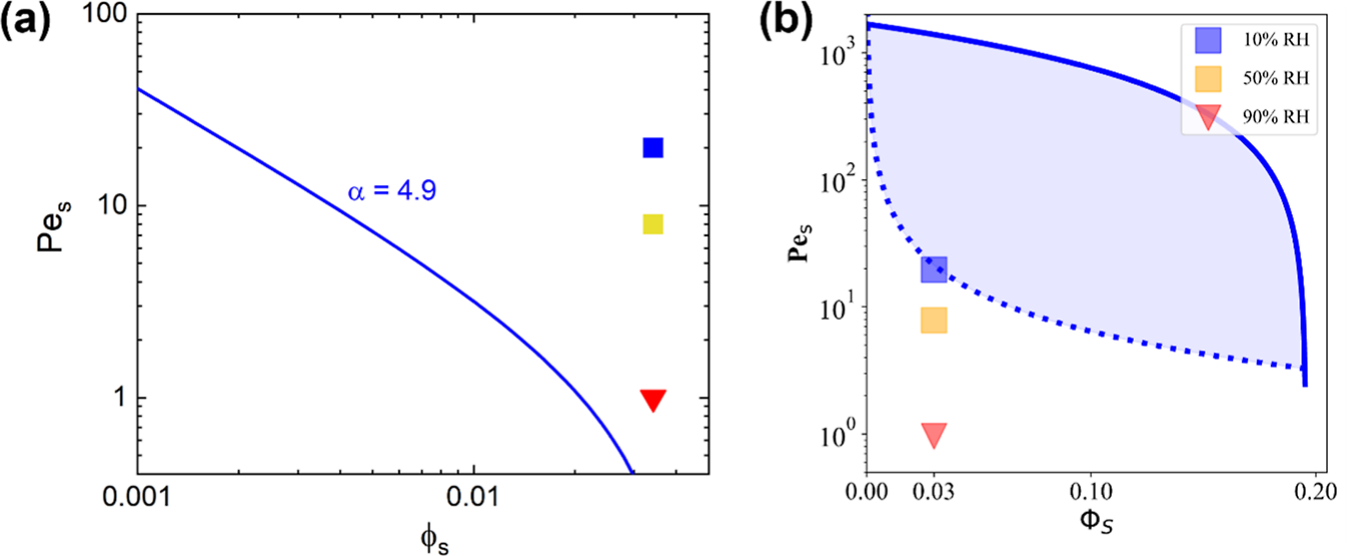
*Pe*_S_ and ϕ_S_ parameter map for binary colloid mixtures at the three environmental conditions studied according to the (a) ZJD model (curve represents the equation α^2^(1 + *Pe*_S_)­ϕ_S_ = 1) and (b) Sear model.

### 3.2 Coating Microstructure

AFM topography maps of the top surface of films formed from binary latex dispersions at three different evaporation rates are presented in Figure 2. Films with *Pe*_S_ = 20 (fast evaporation rate, Figure 2a left column) present a superstructure of small particles arranged into a honeycomb array, which covers the top film surface. This superstructure is templated by the large particles which lay at the bottom of the observed cavities.^21^ For *Pe*_S_ = 8 (medium evaporation rate, Figure 2a center column), a superstructure of small particles, with a comparable height to that obtained at fast evaporation rate, is also observed at the top film surface. The films formed with *Pe*_S_ = 1 (slow evaporation rate, Figure 2a right column) present different surface morphologies. Small particles can be seen forming aggregates in a matrix of coalesced large particles. Aggregation and coalescence processes are enhanced in this case, as the film formation process takes place at a temperature above their glass transition temperature and at 90% RH, the process takes approximately 4 days. A prolonged drying time can result in a much larger number of particle–particle collisions and an increase in the probability of these clustering together.

**Figure 2:**
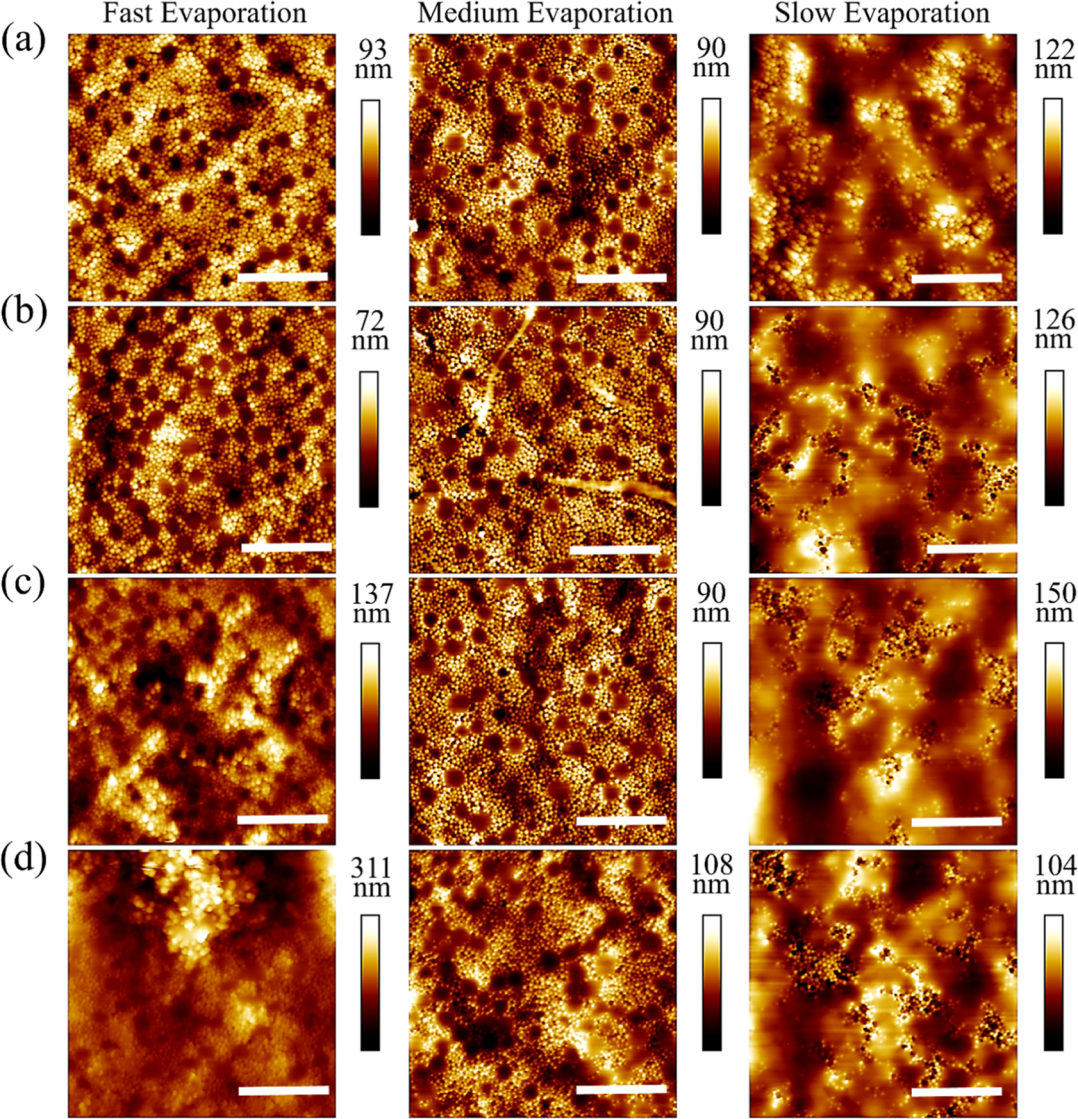
3 μm × 3 μm AFM topography maps of the top surface of bimodal colloidal films formed at 25 °C and three different relative humidities: 10% (fast), 50% (medium), and 90% (slow). (a) Corresponds to the films without added NiPc, (b) 0.1 wt % NiPc, (c) 0.5 wt % NiPc, and (d) 1 wt % NiPc. Scale bars represent 1 μm.

The impact of the addition of NiPc to the film formulation on the film microstructure is dependent on the evaporation rate used. While the top surface of films formed at 10% RH remains fully covered by small particles in the presence of NiPc, increasing its amount induces the formation of additional particle aggregates (Figure 2b–d, left). The presence of these aggregates has a stark effect on the roughness of the films formed at 10% RH, significantly increasing with NiPc concentrations, as shown in Figure 3a. This effect seems to be also present in the films formed at 50% RH but to a lesser degree. The height of the surface superstructures observed, local trough to peak height, at 10% RH and 50% RH appears to have a trend to diminish with increasing NiPc concentration. This observation would agree with the aggregate formation and fewer small particles available to form superstructures, but we cannot draw definitive conclusions on this point because of sample heterogeneity. No significant changes in the surface structure determined by AFM were observed for films formed at 90% RH when the NiPC concentration was varied.

**Figure 3:**
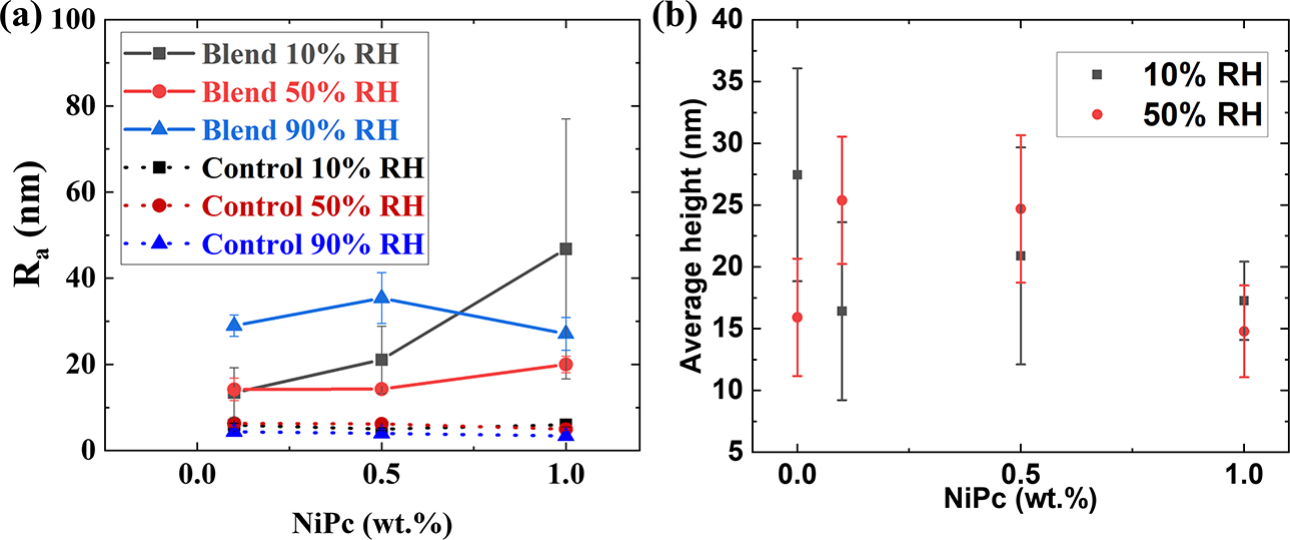
Surface parameters for the coatings under study: (a) roughness (*R*_a_) and (b) mean height of small particle superstructures. Control samples contain only large latex particles blended with 0.1, 0.5, or 1 wt % NiPc.

SEM imaging of cross sections of binary colloidal films without NiPc confirms the observations made via AFM, as shown in Figure 4a. For films formed at 10% and 50% RH, an accumulation of small particles at the top surface can be seen, whereas for 90% RH, this accumulation is not clearly visible. Similar observations can be drawn from films containing 1 wt % NiPc, with the slower evaporation rate resulting in large aggregates of small particles across the film thickness. Small particles have a significantly larger surface area-to-volume ratio, which increases the probability of NiPc adsorption, driven by hydrophobic interactions. This, together with a lower zeta potential for the small particles (Figure S5), results in a stronger tendency for them to aggregate during film formation.^22^ Moreover, slower evaporation rates and, therefore, longer drying times will result in more particle–particle collisions and further aggregation.

**Figure 4:**
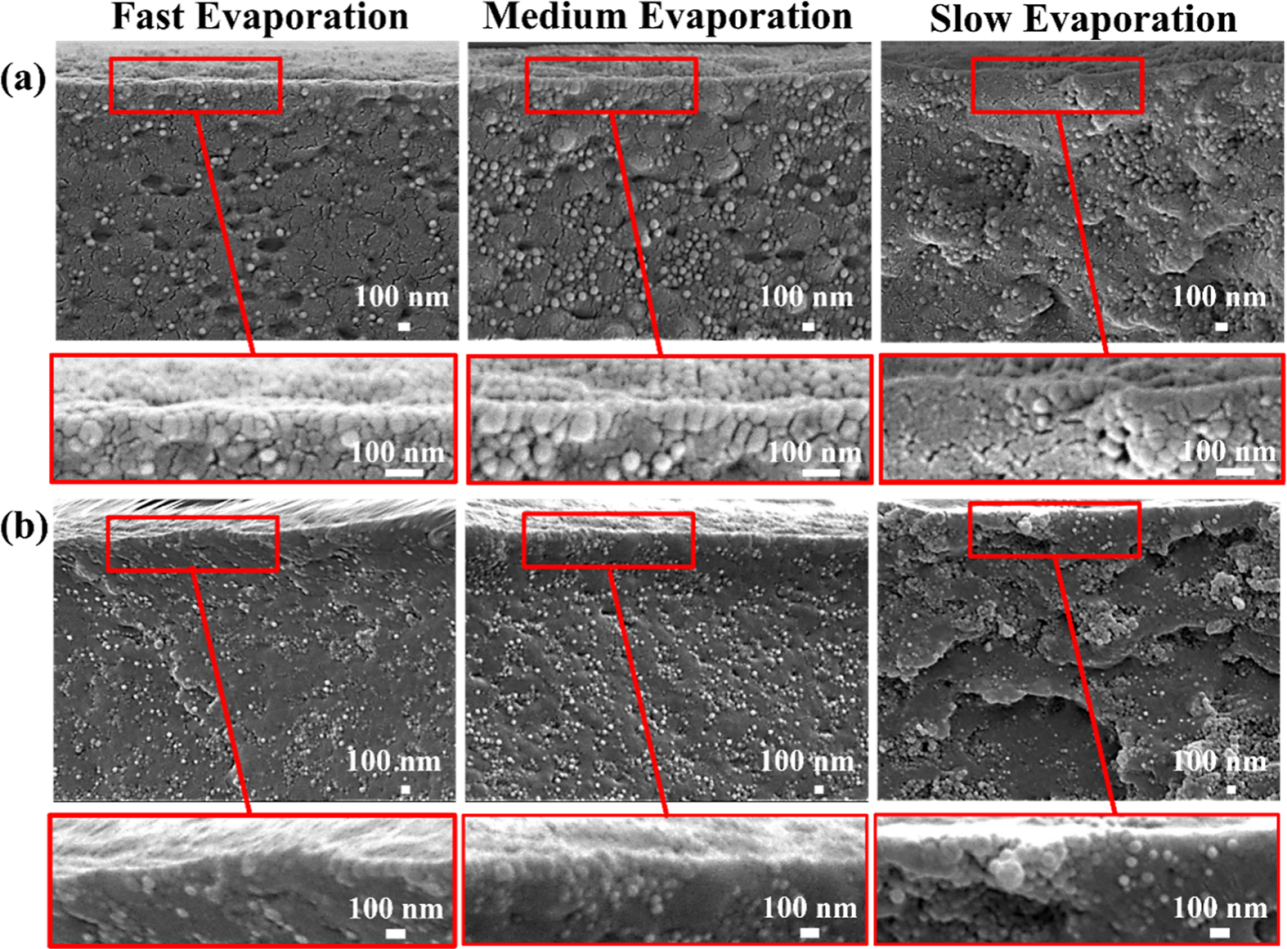
SEM images of cross-section of dried films prepared from blends of large and small particles at 10% RH (fast), 50% RH (medium), and 90% RH (slow): (a) without NiPc and (b) with 1 wt % NiPc.

### 3.3 Small Molecular Additive Distribution

The next question we address is whether the observed changes in microstructure for different RHs and NiPc concentrations impact the distribution of the latter within the films. To answer this question, we acquired cross-sectional confocal fluorescence microscopy images of the NiPc-containing films. In films dried at 10% and 50% RH, a horizontal line of higher intensity fluorescence is clearly observed at the top of the films for all three NiPc concentrations (Figure 5, left and center columns). It is important to note that the red horizontal bright line is not observed in control films made of only large particles (Figure S6). A control film for the small particles could not be prepared, as these do not form a film under the environmental conditions that we are investigating in this work. Therefore, the measurements presented here are a clear demonstration of the accumulation of NiPc at the top of the films when evaporating at 10% or 50% RH. In films dried at 90% RH (Figure 5, right column), the NiPc distribution changes significantly and appears concentrated on the lower part of the film. The magnitude of the background scatter signal emitted by the glass substrate is consistent across films formed at different evaporation rates. However, slow-drying films have a fluorescence signal heavily concentrated on the lower part of the film, masking the substrate contribution.

**Figure 5:**
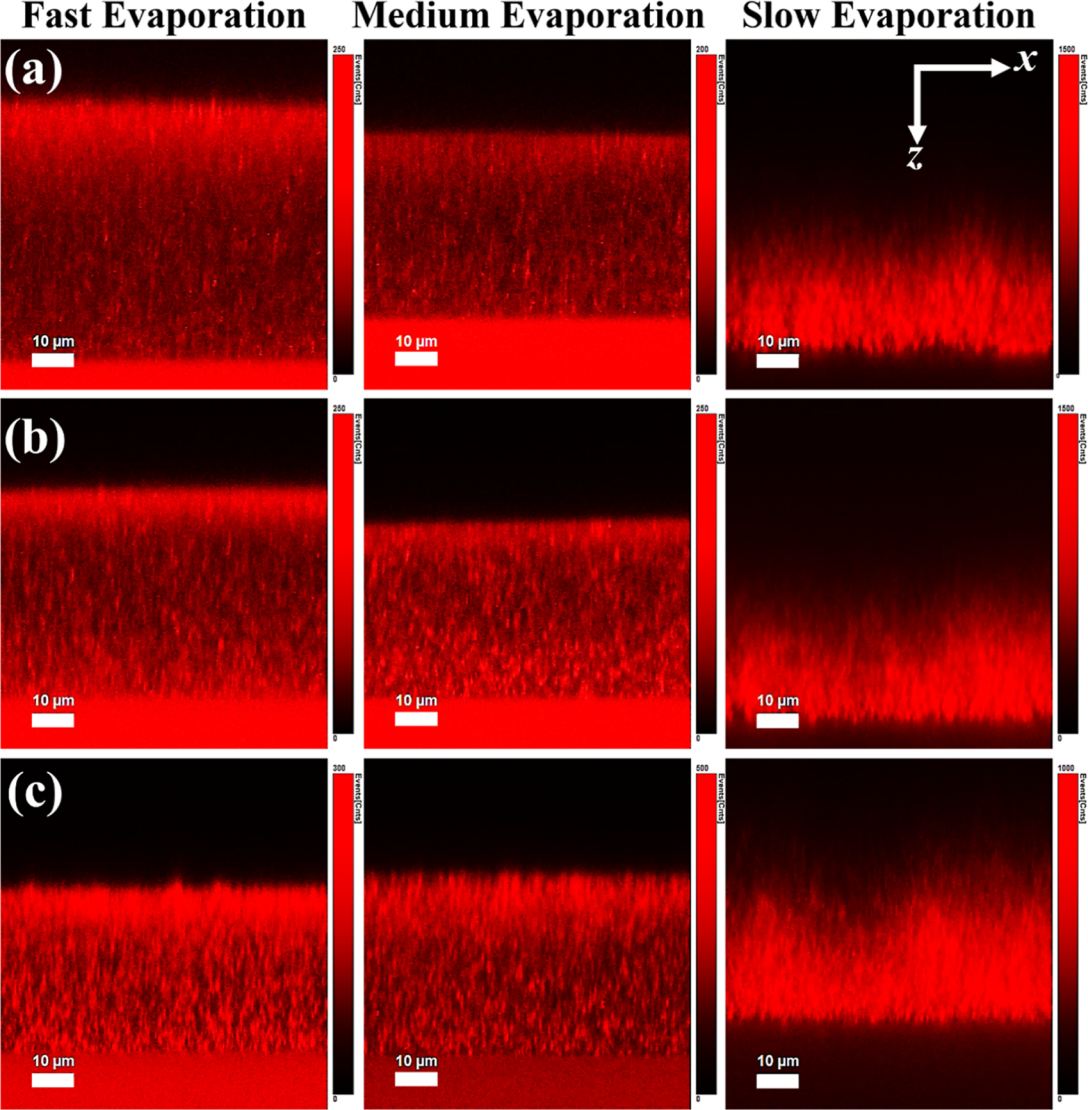
Cross sections of films formed from a binary mixture of large (ϕ_L_ = 0.7) and small particles (ϕ_S_ = 0.3) obtained by confocal fluorescence microscopy with different NiPC concentrations: (a) 0.1 wt %, (b) 0.5 wt %, and (c) 1 wt %. Bright horizontal intensity lines at the film’s top surface observed in samples dried under low and medium humidities indicate NiPc is located at the upper part of the film.

Trends observed in the 2D confocal maps are confirmed by the integrated one-dimensional intensity profiles presented in Figure 6. A prominent peak indicating an enrichment of NiPc at the upper part of the film is consistently detected for the three NiPc concentrations studied in films formed at both 10% and 50% RH conditions. This observation, together with the fact that at these RHs small particles accumulate at the top of the coatings, indicates that small particles play a key role in the final NiPc distribution, as we discuss in more detail later.

**Figure 6:**
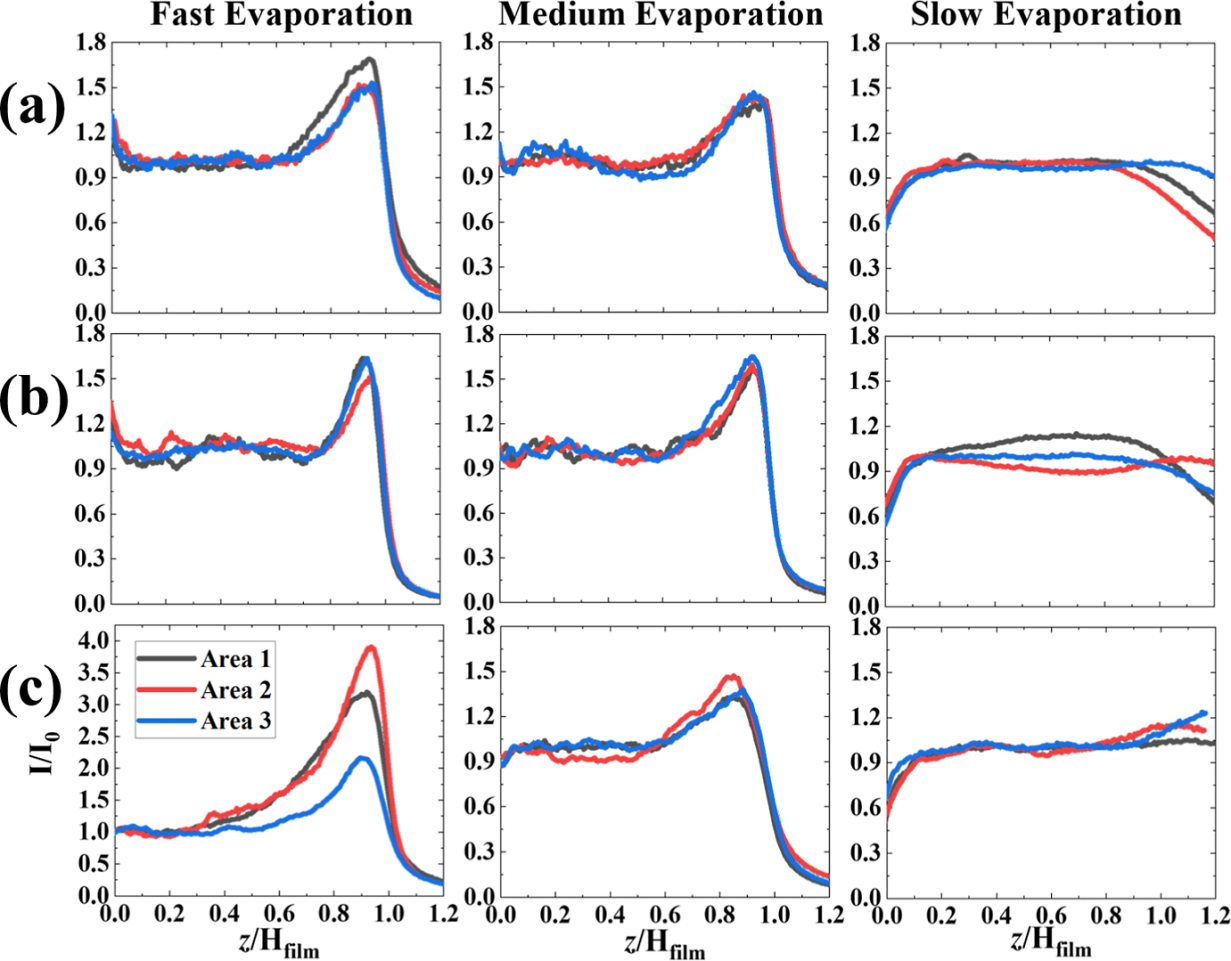
1D intensity profiles obtained from confocal fluorescence microscopy 2D maps as a function of the ratio of the vertical coordinate (*z*) over the full film height. (a) 0.1 wt % NiPc, (b) 0.5 wt % NiPc, and (c) 1 wt % NiPc. The intensity is normalized by the bulk intensity—plateau value within the film—and corrected for depth dependence.

In films dried at 90% RH, NiPc appears to accumulate close to the substrate at the bottom. We note that these films did show large aggregates of small particles in our AFM and SEM investigations, which had sedimented, carrying the NiPc adsorbed on their surfaces with them. Figure 7 compares the normalized intensity of the NiPc peak detected at the top film region as a function of evaporation rate and NiPc concentration. This peak intensity does not seem to depend on the evaporation rate or the concentration of NiPc on its own, except for films containing 1 wt % NiPc formed at 10% RH. The stronger peak intensity is likely to be the result of an enhanced trapping of small particles and/or NiPc clusters at the air/water interface because of the faster evaporation rate. However, control experiments in films containing only the large particles and NiPc do not show a significant accumulation of NiPc at the upper part of the coatings (see Figure S6), and therefore, NiPc clusters at the air/water interface are unlikely.

**Figure 7:**
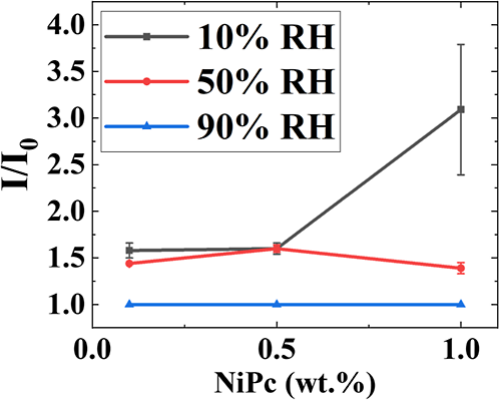
Peak intensity detected at the top of the films, normalized by the bulk intensity, as a function of NiPc concentration at three different evaporation rates.

### 3.4 Small Molecule Adsorption on Polymer Particles

The film characterization presented so far suggests that NiPC is adsorbed mostly on the small particles, and these are carrying the additive with them. As the surface chemistry of the large and small particles is not identical, the next step is to determine whether there is actually a preferential adsorption of NiPc on the small particles. In order to investigate this, two dispersions containing either small or large particles were prepared in a way in which the total particle surface area and NiPc concentration were matched. Details of the calculations can be found in the Supporting Information. After preparing these dispersions, they underwent ultracentrifugation, and the supernatant was analyzed using UV–vis spectroscopy, as shown in Figure 8. The results provided the concentration of unbound NiPc, enabling us to compare the values of NiPc bound to either large or small particles. This concentration turned out to be almost the same for both systems (see Table S2). Therefore, there is no preferential adsorption of NiPc into one of the particle populations. However, in the blends that were used to prepare our films, the total surface area of the small particles is significantly larger than that of the large particles. It is indeed more than double, going from 2.75 × 10^3^ cm^2^ for the large particles to 5.83 × 10^3^ cm^2^ for the small particles (see Supporting Information for details). Therefore, it is expected that a much larger number of NiPc molecules will be adsorbed on the surface of the small particles and transported with them to the upper part of the film for the fast and medium evaporation rate scenarios.

**Figure 8:**
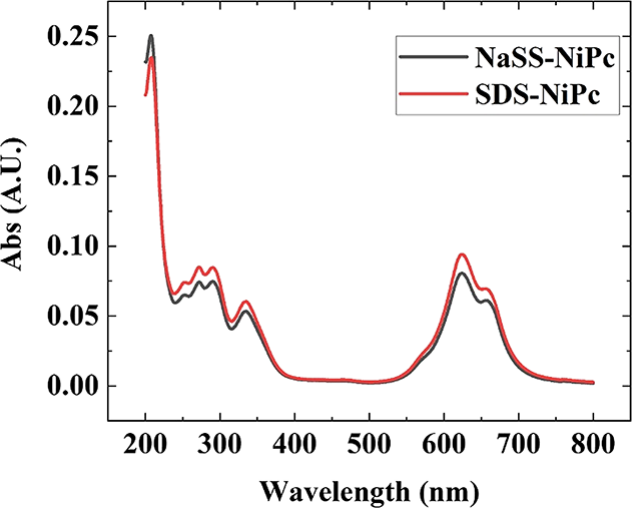
UV–vis absorption spectra of the supernatants extracted from NaSS and SDS ultracentrifuged samples in the presence of NiPC.

### 3.5 Small Molecule Additive Release

Water soaking tests were carried out to assess the impact of film formation conditions and the coating microstructure on NiPc release. UV–vis spectroscopy was used to determine the NiPc released into water in contact with the coating, and its accumulated value over time is plotted in Figure 9 for the systems under study. All the samples show a rapid early release within the first 5–10 min and a slower sustained phase afterward, in most cases achieving controlled release with a constant slope.

**Figure 9:**
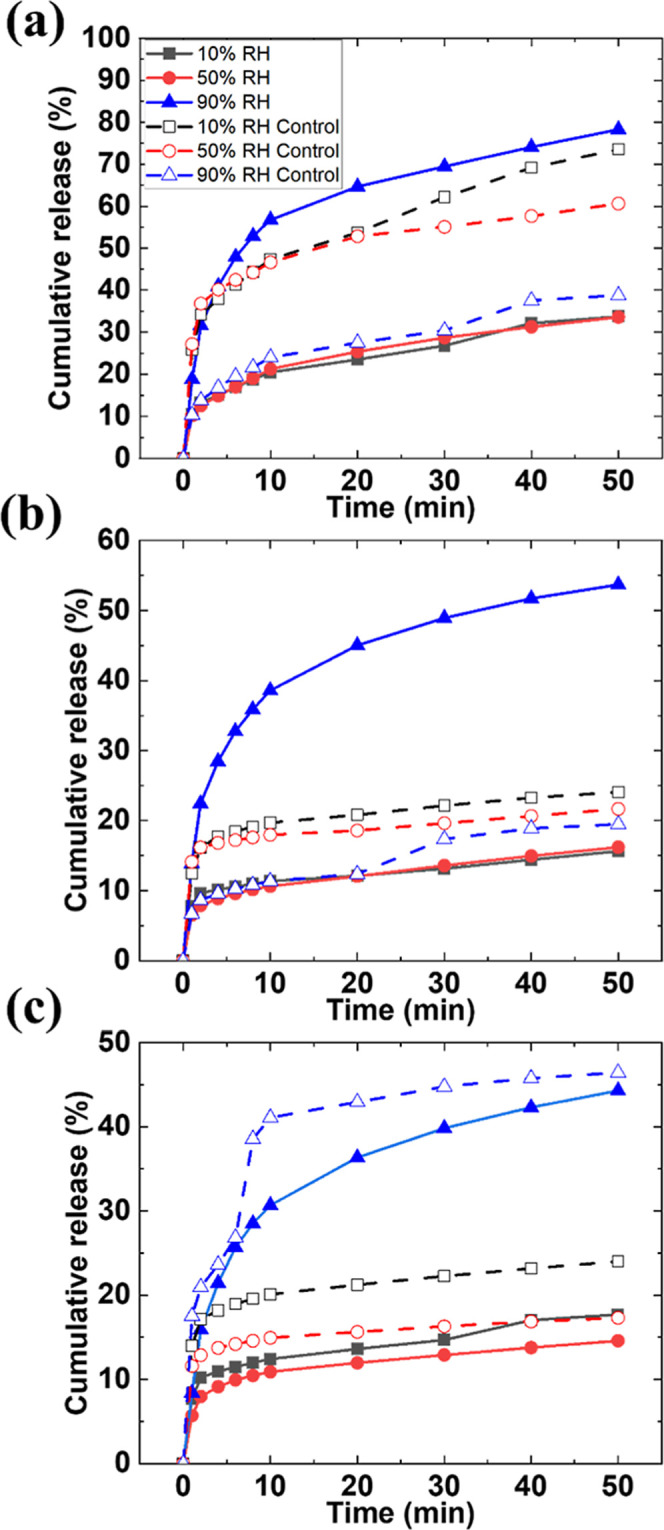
Cumulative NiPc release profiles upon soaking in water of bimodal and control (only large particles) coatings containing: (a) 0.1 wt % NiPc, (b) 0.5 wt % NiPc, and (c) 1 wt % NiPc.

We will focus first on the short-term release, i.e., the cumulative NiPc after the initial 10 min (Figure 10). We have shown how in bimodal films there is an accumulation of NiPc in the vicinity of the top interface for fast and medium evaporation rates (Figure 6). This, together with the enhanced surface roughness when compared to control samples (Figure 3), should enhance the accessibility to NiPc molecules by water and their release from the coatings over short times. However, in most cases, the control systems release a significantly larger amount of NiPc in these initial stages when compared with the bimodal blends, with this difference being larger at lower NiPc concentrations.

**Figure 10:**
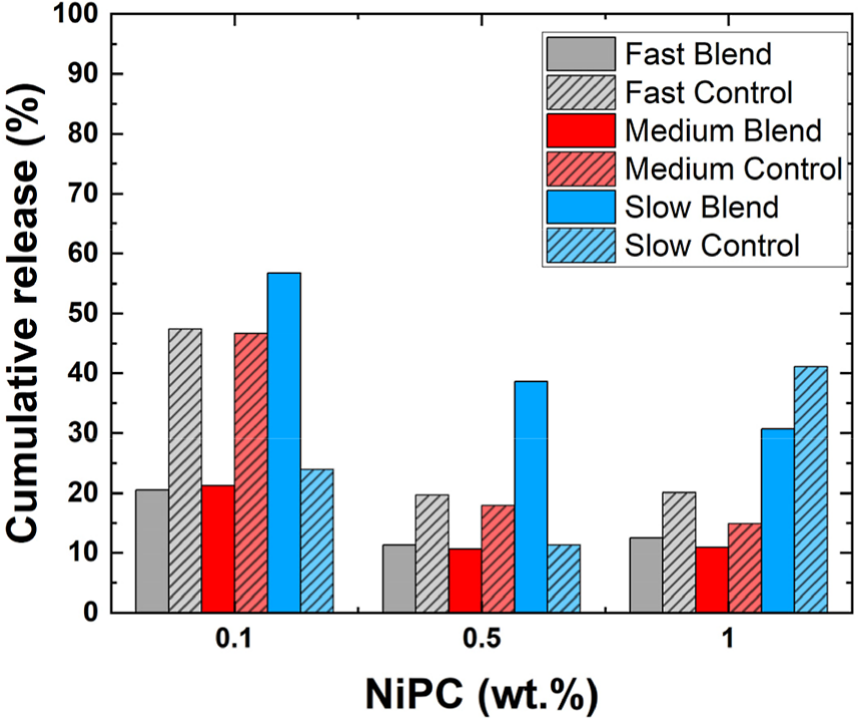
Cumulative NiPc release from bimodal blend films and control samples after 10 min across different drying rates (fast, medium, and slow) and NiPc concentrations (0.1, 0.5, and 1 wt %).

This apparent contradiction suggests that other factors are overriding the effects of the surface roughness and NiPc distribution. One possibility is that the increased porosity or heterogeneity in the bimodal films creates regions where NiPc is more trapped within the polymer matrix, reducing its mobility and slowing its diffusion into water. In contrast, the more uniform control films may allow more continuous diffusion pathways, facilitating a faster initial release. Additionally, the local environment in bimodal blends, with an increased surface area due to reduced small particle size, may enhance hydrophobic interactions with NiPc, slowing down its desorption. This effect seems more pronounced at lower NiPc concentrations, where fewer molecules are available to saturate the local polymer particle network, making the trapping effect more significant.

Overall, the small-on-top microstructures of these systems allow for a more controlled release of small molecules from the coatings. This feature could be desirable for a range of applications in the dental, marine, and medical sectors when the coating needs to deliver a functional agent gradually over time.

Exceptions to this trend are the bimodal blends film formed at a slow evaporation rate at 0.1 and 0.5 wt % of NiPc. These not only show a more significant short-term NiPc release when compared to their control counterparts, but they also present the largest long-term release of all systems under study (Figure 9). Considering the microstructure of these systems, which do not present a small-on-top structure or NiPc accumulation near the top coating interface, this is unexpected. Bodmeier and Paeratakul studied the difference in drug release from colloid polymer films when stored at different relative humidities.^23^ They found that films stored at high humidity not only released the drug faster when soaked in water but also exhibited a greater overall release compared to films stored at lower humidities. We could be observing a similar phenomenon in our samples. The presence of extra moisture in our coatings dried at high humidity could lead to some degree of polymer plasticization, facilitating the ingress of water and, therefore, NiPc release.

The NiPc profiles (Figure 9) were fitted to the Korsmeyer–Peppas (KP) model^24^ to extend the study from the short to the medium-term release

$$\frac{M_{t}}{M_{\infty }}=kt^{n}$$

where *M*_*t*_ is the mass of NiPC released at time *t*, *M*_∞_ is the total amount of NiPc in the system, *k* is the kinetic constant, and *n* is the release exponent. The *k* parameter provides insights into the rate of NiPc release, while the *n* exponent characterizes the mechanism of release. The individual KP model fittings are provided in Figures S7–S9.

As seen in Figure 11, the release constant *k* for the bimodal films is generally lower than that for the control films in most cases - except for the slow evaporation rate scenario at lower NiPc content. This is expected from our short-term release observations and the release profiles presented in Figure 9. For all samples, *n* is significantly under 0.5, indicating that NiPc undergoes Fickian diffusion during the release test.^25^ In many cases, *n* is significantly smaller for the control films compared to the bimodal blends. This could suggest a difference in the polydispersity of the pores that the NiPc diffuses through when moving within the films.

**Figure 11:**
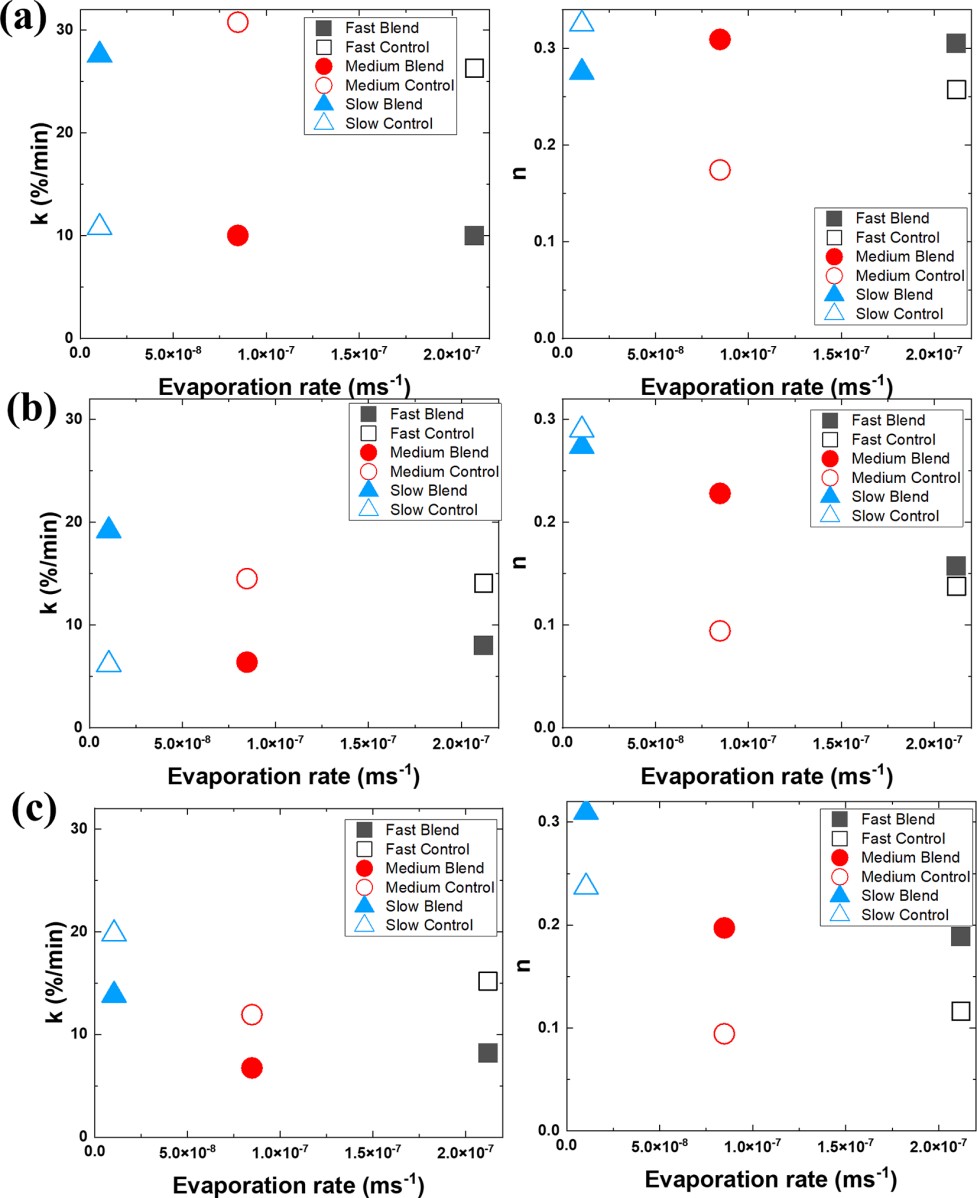
Parameters obtained from fitting the release profiles using the Korsmeyer–Peppas models. On the left column, the release rate constant *k*. In the right column, release exponent *n*. The NiPc concentrations in the coatings are (a) 0.1 wt %, (b) 0.5 wt %, and (c) 1.0 wt %.

## 4 Conclusions

Our study demonstrates that particle size segregation during the drying of bimodal colloidal blends can be successfully harnessed to control the distribution of small-molecule additives in coatings. Using NiPc as a case study, we showed how it accumulates at the top part of the film when dispersed in bimodal latex blends and film formed at fast and medium evaporation rates.

By combining confocal fluorescence, scanning electron, and atomic force microscopy, we proved that the NiPc distribution is correlated with the presence of small particles in the films’ upper region. This was not the case in control films containing only one size of particles and NiPc, which presented a homogeneous distribution of the additive across their thicknesses. Preferential adsorption tests by means of UV–vis spectroscopy revealed that NiPc has no preferential adsorption onto either large or small particles. However, the significantly greater total surface area of small particles in the blend means that they serve as the main carriers of NiPc.

Release tests show that bimodal films generally provide more controlled and sustained release of NiPc compared to control films of only large particles. While short-term (initial 10 min) release is lower for bimodal blends, exceptions occur at slow evaporation rates and low NiPc concentration, where higher release rates and overall release are observed. These are likely due to increased polymer plasticization at high humidity. Fits to the Korsmeyer–Peppas model indicate that NiPc in all systems predominantly undergoes Fickian diffusion, and differences in release exponent *n* suggest variations in pore polydispersity across the different systems under study.

Our research demonstrates that controlling particle size distribution and evaporation rate enables tuning of the spatial distribution of small molecule additives in polymer coatings, leading to tailored release profiles. This approach holds promise for applications requiring sustained or targeted delivery of functional agents, such as in dental, marine, or medical coatings.

## Supplementary Material



## Notes

Determination of evaporation rates, UV–vis absorbance calibration, DSC analysis of dried copolymers, Péclet number calculations, diameter and zeta potential changes, control confocal fluorescence measurements, surface area calculations in the dispersions used for adsorption analysis, and surface area calculations in dispersions used for forming films (PDF)
