# Supplementary material for: Harnessing Particle Size Segregation To Tune Molecular Additive Distribution in Coatings
Source: Ind Eng Chem Res. 2026 Jan 6;65(2):1241–51. doi: 10.1021/acs.iecr.5c04051 (PMC12828723; doi:10.1021/acs.iecr.5c04051)
Supplement: Supplementary file 1 [file ie5c04051_si_001.pdf]

Supporting Information

Harnessing Particle Size Segregation to Tune Molecular Additive Distribution in Coatings

Huyen Le[1], Timothy J. Murdoch,[1] Aitor Barquero,[2] Radmila Tomovska,[2,3] and Ignacio Martin-Fabiani[1]*

[1] Department of Materials, Loughborough University, Leicestershire LE11 3TU, UK

[2] POLYMAT and Departamento de Química Aplicada, Facultad de Químicas, University of the Basque Country, UPV/EHU, Joxe Mari Korta Zentroa, Tolosa Hiribidea 72, Donostia-San Sebastian 20018, Spain

[3] IKERBASQUE, Basque Foundation for Science, Maria Diaz de Haro 3, 48013 Bilbao, Spain

*E-mail: i.martin-fabiani@lboro.ac.uk

Determination of evaporation rates

To measure the water mass loss over time, 200 μL of deionized water were cast on a glass coverslip (18 mm × 18 mm). After recording the mass of water at the initial time (t0), the coverslip was placed inside an environmental chamber set up at 25 °C and either 10%, 50% or 90% RH. To record mass during drying at 10 minutes intervals minimising the impact of evaporation outside the environmental chamber, the procedure was as follows: i) wait for 10 minutes, take the coverslip out, record mass, ii) place a fresh 200 μL water sample in the environmental chamber, wait for 20 minutes, take it out, record mass, iii) repeat step ii) increasing the interval 10 minutes every time until water has fully evaporated.

The resulting date enabled the calculation of the evaporation rate $\dot{E}$ (in ms^{-1}):

$$\dot{E} = \frac{-(\frac{\Delta m}{\Delta t})}{A \times \rho_W}$$

where $\frac{\Delta m}{\Delta t}$ is the evolution of mass of evaporated water over time, A is the surface area of the sample ($A = 3.24\times10^{-4}$ m^2) and ρ_W is the density of water ($\rho_W = 1 \times 10^6 gm^{-3}$).

For the fast evaporation condition, the water fully evaporated within the first hour of experiments, whereas under slower evaporation conditions, measurements were collected over

at least an hour. $\frac{\Delta m}{\Delta t}$ was calculated by performing a least-squares fitting to the data (Figure S1), and the slope was used to determine the evaporation rate. The calculated values were 2.12 × 10^{-7} m/s (fast), 8.47 × 10^{-8} m/s (medium), and 1.04 × 10^{-8} m/s (slow). During the evaporation experiments, the water mass loss is linear with time. It is therefore expected to be roughly proportional to 1 – RH.

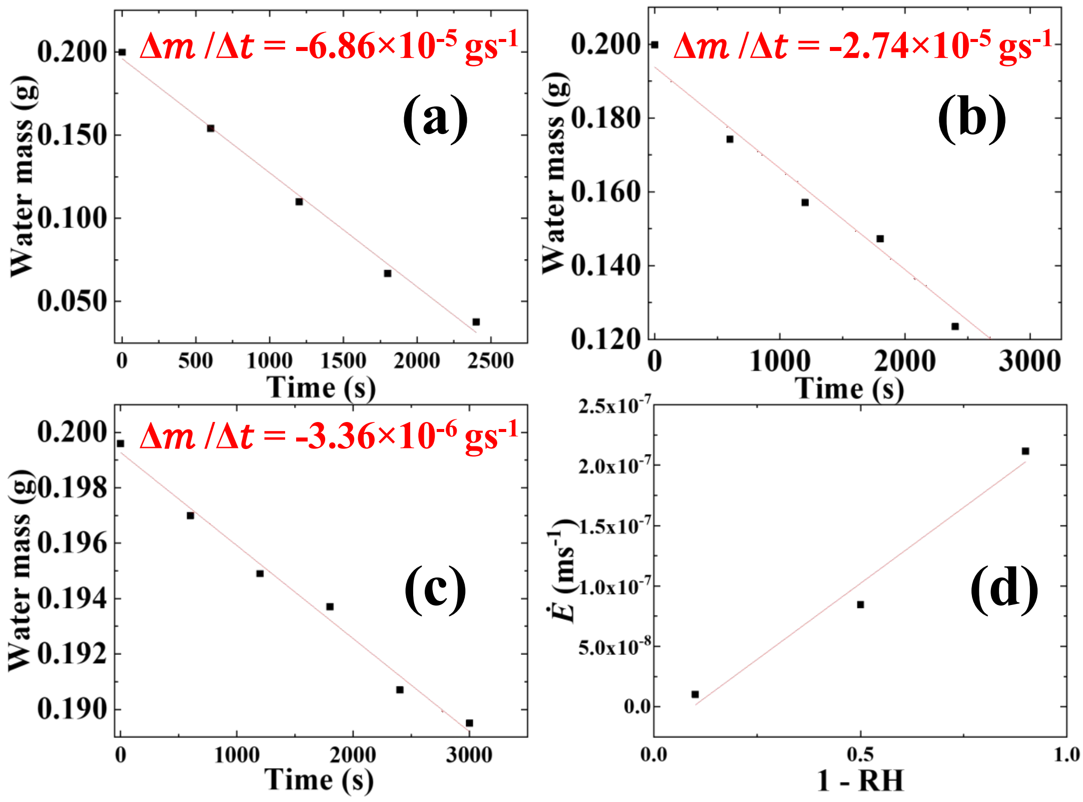

Figure S1. Rate of mass of water loss over time under a) fast drying rate at 10% RH, b) medium drying rate at 50% RH, c) slow drying rate at 90% RH, and d) shows a proportional relationship between evaporation rates and 1-RH.

UV-Vis absorbance calibration

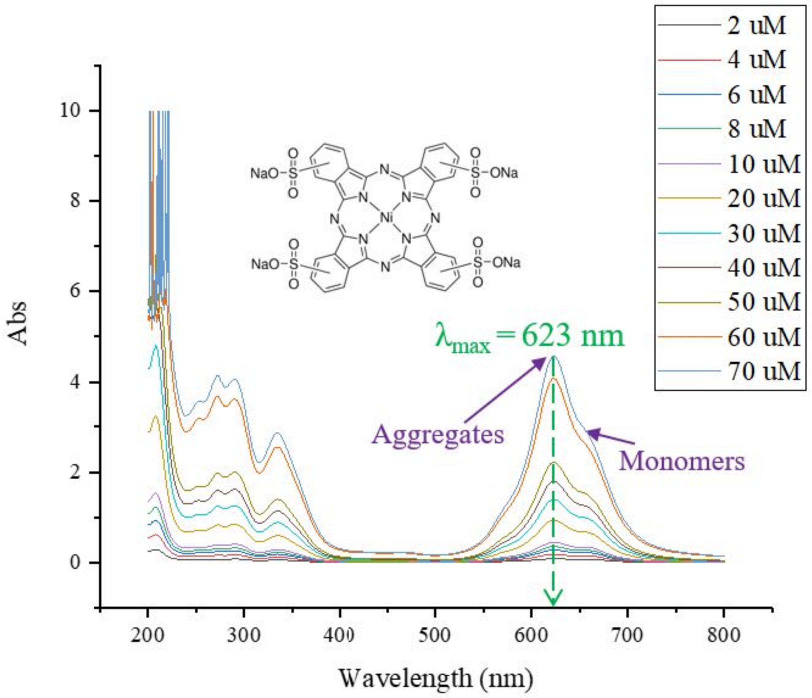

Figure S2. UV-Vis absorption spectra of NiPc during the determination of maximum absorption values at concentrations 2-70 µM, at λ_{max} = 623 nm.

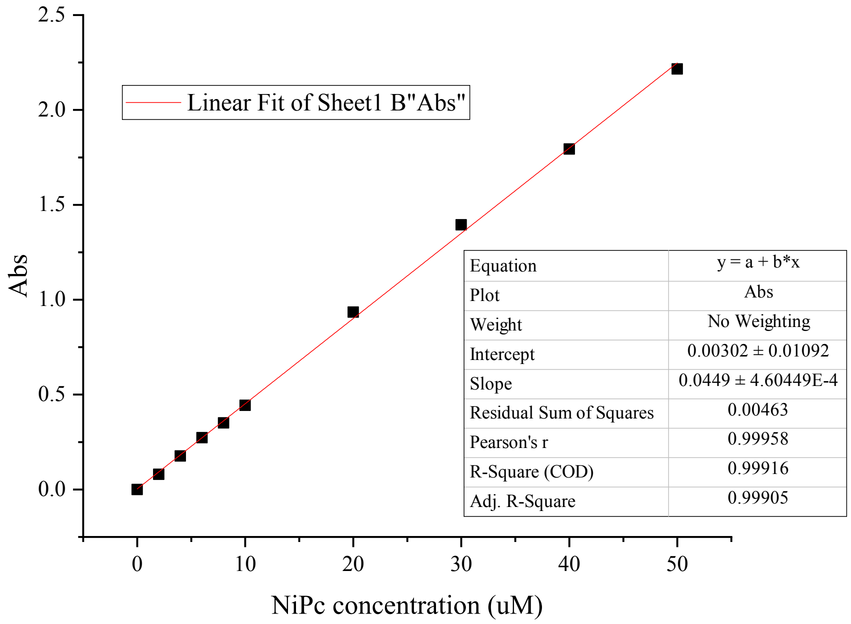

Figure S3. Determination of the molar absorption coefficient of NiPc using a calibration curve extracted from the maximum absorption values of NiPc between 2-70 µM, at λ_{max} = 623 nm.

DSC analysis of dried copolymers

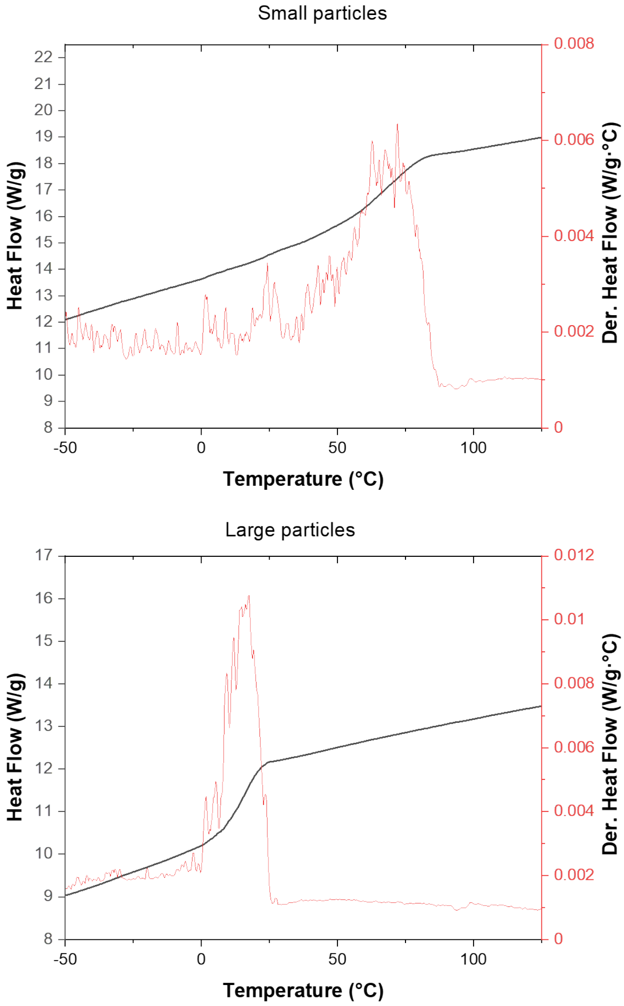

Figure S4. Temperature-dependent heat flow and its derivative for small and large polymer particles.

Péclet number calculations

To calculate the diffusion coefficient of the particles, D, the Stokes-Einstein equation was used:

$$D = \frac{k_b T}{6\pi\eta r}$$

where k_B is the Boltzmann constant ($k_B = 1.38\times10^{-23}$ JK^{-1}), T is the temperature in Kelvin, η is the viscosity of the fluid ($\eta = 1\times10^{-3}$ Pa s), and r is the hydrodynamic radius of the particle. The diffusion coefficients of small and large latex particles at 25°C were calculated as 6.69×10^{-12} m^2s^{-1} and 1.35×10^{-12} m^2s^{-1}, respectively. With these values and the measured evaporation rates, we can calculate the Péclet numbers for different film formation conditions:

$$Pe = \frac{\dot{E}H}{D}$$

where H is the wet film thickness, $H = 6.17\times10^{-4}$ m. Calculated Péclet numbers for large and small particles under different relative humidities are presented in Table S1.

Table S1. Summary of film forming conditions and corresponding values of evaporation rate and Peclet numbers (rounded to the nearest integer), at 25 ^{o}C.

**Film formation conditions**	$\dot{E}$ **(ms^{-1})**	Pe_S	Pe_L
25C, 10% RH	2.12×10^{-7}	20	100
25C, 50%RH	8.47×10^{-8}	8	38
25C, 90%RH	1.04×10^{-8}	1	5

Diameter and zeta potential changes

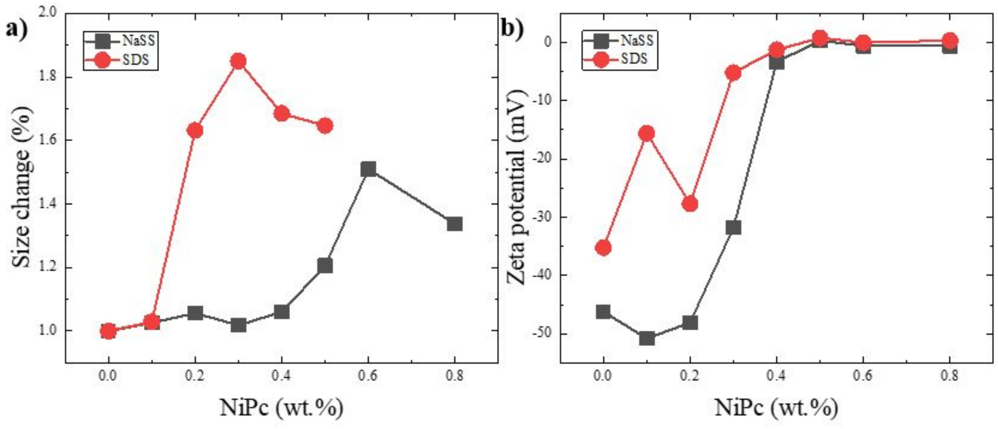

Figure S5. Effects of adding NiPc to each particle population on a) particle diameter and b) zeta potential. NaSS tag corresponds to large particles and SDS tag to small particles.

Control confocal fluorescence measurements

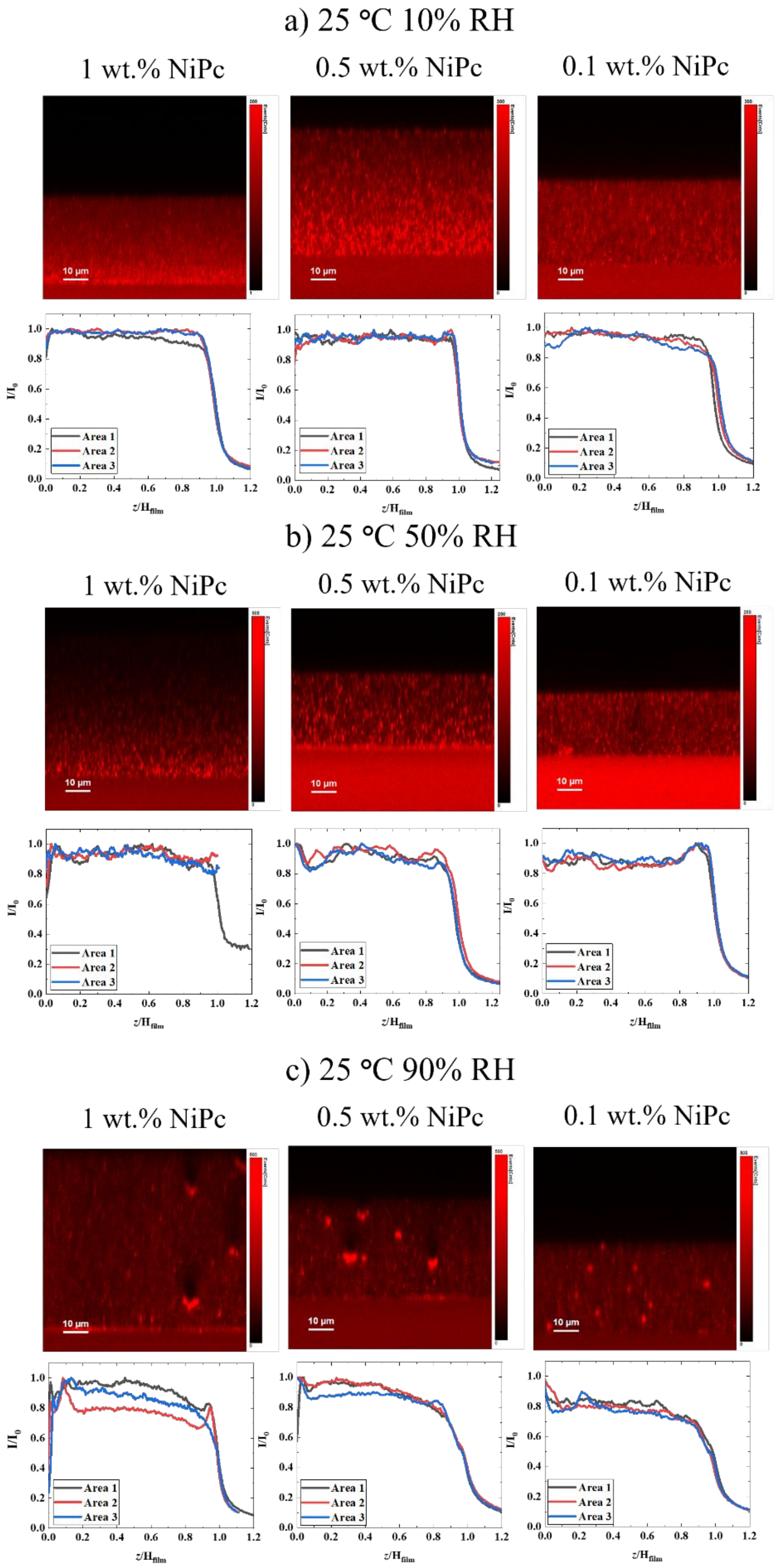

Figure S6. Confocal fluorescent microscopy scans and 1D profiles of control samples containing only large latex particles blended with 0.1, 0.5 and 1 wt.% NiPc film formed at 25 °C and a) 10% RH, b) 50% RH and c) 90% RH.

Surface area calculations in the dispersions used for adsorption analysis

Based on the hydrodynamic diameter obtained by DLS, we can calculate the surface area of a single small particle, yielding:

$$A_{large} = 4\pi\left(\frac{267\ x\ 10^{-7}cm}{2}\right)^2 = 2.24\ x\ 10^{-9}cm^2$$

$$A_{small} = 4\pi\left(\frac{54.1\ x\ 10^{-7}cm}{2}\right)^2 = 9.19\ x\ 10^{-11}cm^2$$

We can also calculate the mass of a single small or large particle, assuming a density 1.14 g cm^{-3}, an weighted average between those of PBA and PMMA:[1]

$$m_{large} = 1.14\ g\ cm^{-3} x \frac{4}{3}\pi\left(\frac{267\ x\ 10^{-7}cm}{2}\right)^3 = 1.14\ x\ 10^{-14}g$$

$$m_{small} = 1.14\ g\ cm^{-3} x \frac{4}{3}\pi\left(\frac{54.1\ x\ 10^{-7}cm}{2}\right)^3 = 9.45\ x\ 10^{-17}g$$

To assess the binding affinity of NiPc to different latex particles, ultracentrifuge experiments were designed to match the total surface area of particles present in each dispersion. The measured volume of the 10 wt.% large NaSS dispersion was 4450 μL, which allows to calculate the number of large particles in the sample as follows:

$$N_{large\ centrifuge} = \frac{0.1\ x\ 4.45\ g}{1.14\ x\ 10^{-14}\ g} = 3.9\ x\ 10^{13}$$

And a total surface area of:

$$A_{large\ cantrifuge} = 3.9\ x\ 10^{13}\ x\ 2.24\ x\ 10^{-9}\ cm^2 = 8.7\ x\ 10^4\ cm^2$$

The measured volume of the 10 wt.% small SDS dispersion was 900 μL and therefore

$$N_{small\ centrifuge} = \frac{0.1\ x\ 0.9\ g}{9.45\ x\ 10^{-17}\ g} = 9.52\ x\ 10^{14}$$

And a total surface area of:

$$A_{small\ centrifuge} = 9.52\ x\ 10^{14}\ x\ 9.19\ x\ 10^{-11}\ cm^2 = 8.7\ x\ 10^4\ cm^2$$

Making the total particle surface area match for both dispersions.

Table S2. UV-Vis absorbance values of corresponding unbound NiPc concentrations determined from the ultracentrifuge supernatant samples.

**Sample**	**Absorbance (A.U.)**	**[NiPc] in supernatant (uM)**	**Bound [NiPC] (M)**
**NaSS-NiPc**	0.081	1.8	0.0112784
**SDS-NiPc**	0.094	2.1	0.0112779

Surface area calculations in dispersions used for forming films

Our bimodal blends contain 70% large particles and 30% small particles in a 10 wt.% solids dispersion. This means that the 200 µl samples (~0.2 g) we cast to form films contain 0.02 g of particle mass, of which 0.014 g are large particles and 0.006 g are small particles.

With this information we can estimate the number of particles and the surface area of each population:

$$N_{large} = \frac{0.014g}{1.14\ x\ 10^{-14}g} = 1.23\ x\ 10^{12}$$

$$N_{small} = \frac{0.006g}{9.45\ x\ 10^{-17}g} = 6.35\ x\ 10^{13}$$

And, finally, the total surface area for each population:

$$S_{large} = 1.23\ x\ 10^{12}\ x\ 2.24\ x\ 10^{-9}cm^2 = 2.75\ x\ 10^3cm^2$$

$$S_{small} = 6.35\ x\ 10^{13}\ x\ 9.19\ x\ 10^{-11}cm^2 = 5.83\ x\ 10^3cm^2$$

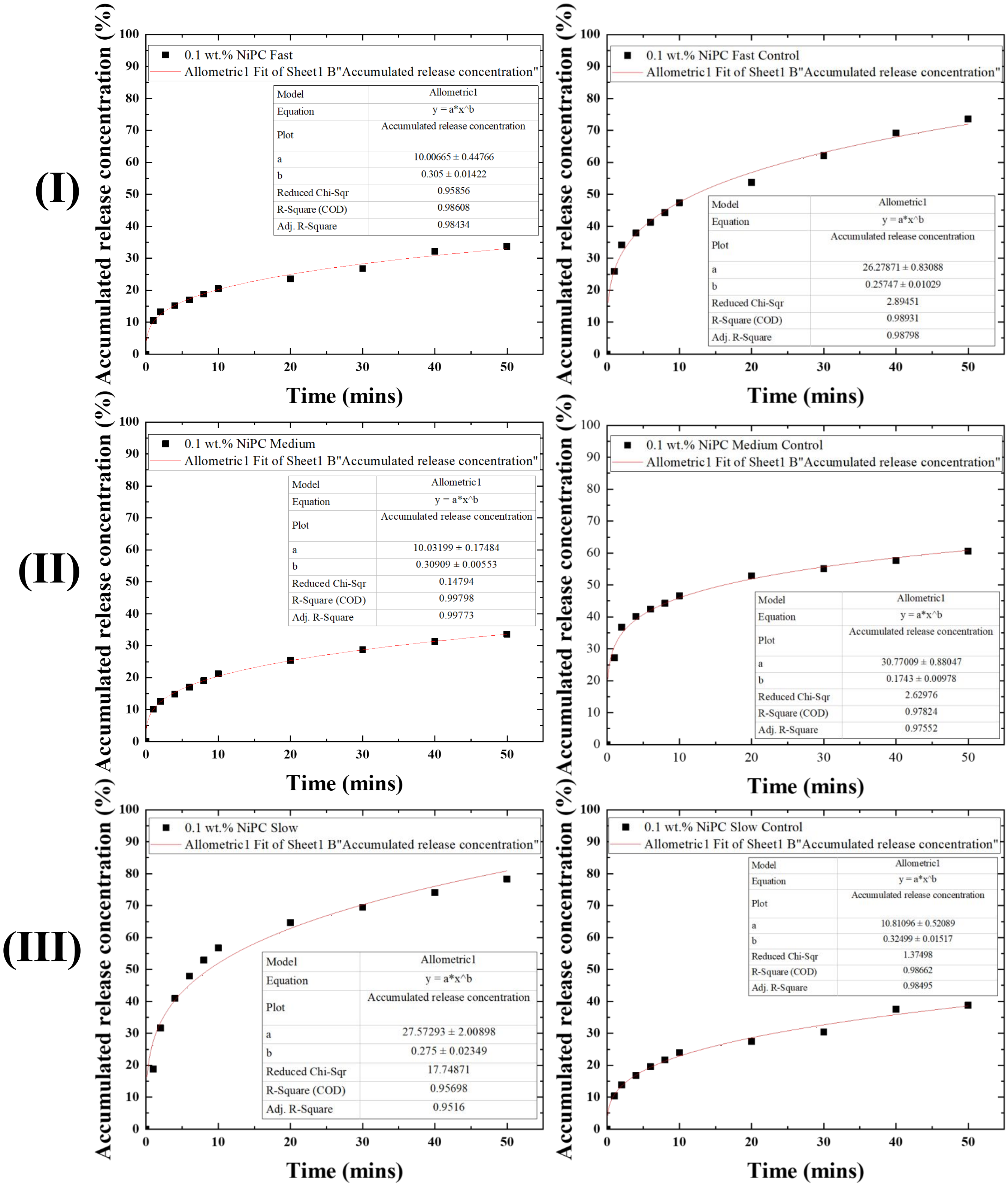

Figure S7. Individual Korsmeyer-Peppas model fittings for 0.1 wt.% NiPc release in (a) NaSS-SDS blend samples and (b) NaSS control samples, categorised by drying rates : I) fast, II) medium, and III) slow.

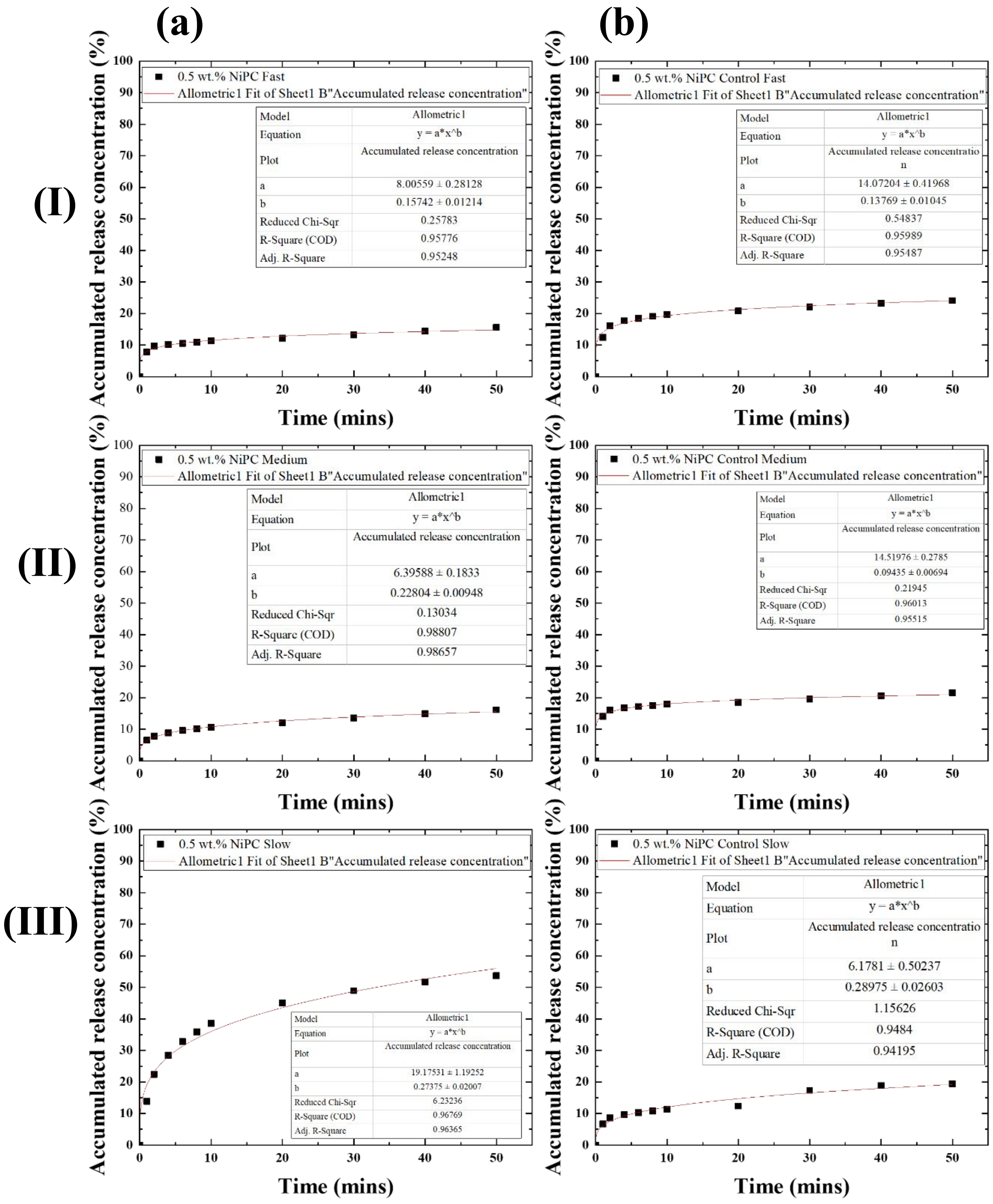

Figure S8. Individual Korsmeyer-Peppas model fittings for 0.5 wt.% NiPc release in (a) NaSS-SDS blend samples and (b) NaSS control samples, categorised by drying rates : I) fast, II) medium, and III) slow.

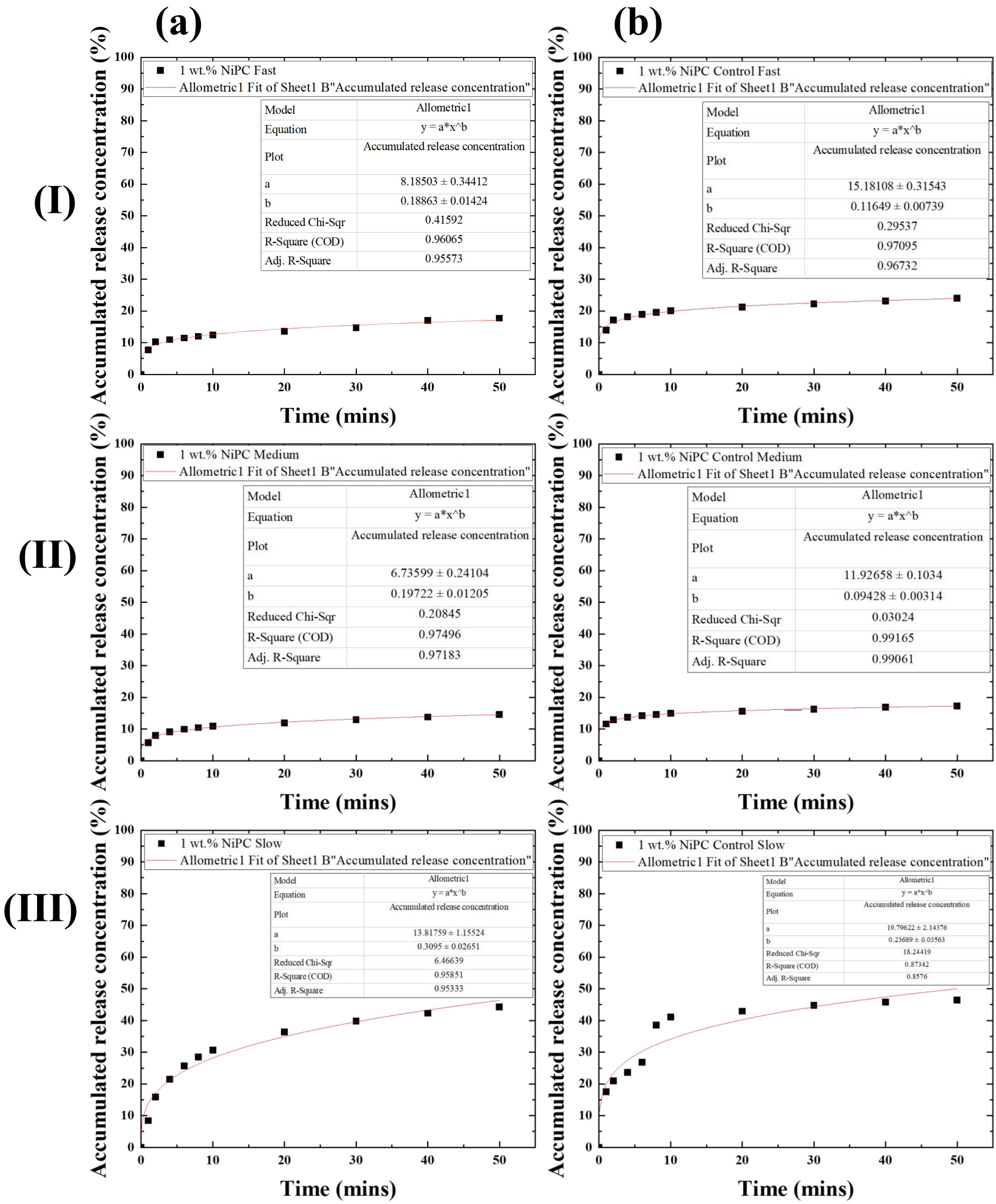

Figure S9. Individual Korsmeyer-Peppas model fittings for 1 wt.% NiPc release in (a) NaSS-SDS blend samples and (b) NaSS control samples, categorised by drying rates : I) fast, II) medium, and III) slow.

References

[1]Mark, J. E., Ed. *Physical Properties of Polymers Handbook*; 2nd ed.; Springer: New York, 2007.
